# A novel method for spatial source localization using ECoG and SEEG recordings in human epilepsy patients

**DOI:** 10.1186/1471-2202-16-S1-P286

**Published:** 2015-12-18

**Authors:** Chaitanya Chintaluri, Daniel K Wójcik

**Affiliations:** 1 Department of Neurophysiology, Nencki Institute of Experimental Biology, Warsaw - 02093, Poland

## 

In recent decades, there has been increasing interest in surgical treatments of patients with pharmacologically intractable epileptic seizures. In some cases, noninvasive methods do not sufficiently localize epileptogenic foci and invasive methods of presurgical evaluation are necessary. In some patients, craniotomy is required, where-in electrodes are placed directly over the cortex - these recordings are referred to as electrocorticography (ECoG). Another invasive method is based on intra-cortical depth electrodes which are stereotactically steered into deep cerebral structure - these are referred to as stereoencephalography (SEEG). Based on these recordings of electrical potentials at corresponding electrode positions, the spatial location of the sources of epileptic activity in the brain, that is to be lesioned, needs to be estimated. Improving the precision of locating these sources - referred to as source imaging, derived from ECoG and SEEG recordings remains a critical goal in the field [1].

Based on our previous studies [2], we propose a new method - kESI (kernel Electrical Source Imaging), which takes into account realistic brain morphology and spatial variations in brain conductivity. kESI can localize multiple sources, and is flexible to arbitrary electrode positions. Therefore this method can be effectively used for a specific patient's case. The core of kESI is in the construction of kernel functions requiring computation of the potentials generated in the brain by numerous basis functions covering the probed volume.

**Figure 1:**
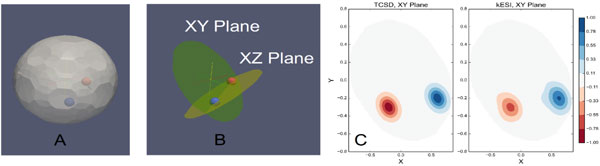
****A**) Shows the VTK rendering of the simplified 3D brain model, the red-blue spheres indicate the placement of a dipole inside the brain**. **B**) Shows the two orthogonal planes passing through the dipole (red-blue spheres) and origin **C**) Reconstruction of sources from simulated ECoG recordings, left:True CSD, right:kESI method (along XY Plane). The RMS difference between True CSD and kESI method, evaluated along XY and XZ planes is 0.248

To test the robustness of the method, we generated ground truth data using a simplified spherical brain model - with uniform conductivity, and placed a dipole source inside it (Figure 1A). The electrodes were placed on the surface of the sphere, and were also placed inside the spherical volume emulating ECoG and SEEG style recordings respectively. The potentials generated at these electrodes were computed using Finite Element Methods (FEM) in FEniCS software, the mesh was generated in gmsh. In kESI, this FEM model was used to compute the potentials generated by the basis functions, and hence obtain the reconstructed sources. To evaluate the accuracy, the root mean squared difference (RMS) values between the ground-truth and the reconstructed sources from kESI was computed in the orthogonal planes passing through the dipole (Figure 1B). This was found to be 0.158 when using 652 electrodes (ECoG & SEEG), and 0.248 when using 100 electrodes (only ECoG) (Figure 1C).
